# Never in mitosis gene A-related kinase 6 promotes cell proliferation of hepatocellular carcinoma via cyclin B modulation

**DOI:** 10.3892/ol.2014.2300

**Published:** 2014-06-30

**Authors:** BIAO ZHANG, HAI ZHANG, DONG WANG, SHENG HAN, KE WANG, AIHUA YAO, XIANGCHENG LI

**Affiliations:** 1 Liver Transplantation Center, First Affiliated Hospital of Nanjing Medical University, Key Laboratory of Living Donor Liver Transplantation, Ministry of Public Health, Nanjing, Jiangsu 210029, P.R. China; 2 Department of Hepatobiliary Surgery, Affiliated Hospital of Jiangsu University, Zhenjiang, Jiangsu 212000, P.R. China

**Keywords:** never in mitosis gene A-related kinase 6, proliferation, cyclin B, hepatocellular carcinoma, cdc2

## Abstract

Never in mitosis gene A-related kinase (Nek) 6 is a recently identified Nek that is required for mitotic cell cycle progression; however, the role and mechanism of Nek6 activity during hepatocarcinogenesis is not well known. The aim of this study was to investigate the potential roles and internal mechanism of Nek6 in hepatocellular carcinoma (HCC) development. In the present study, Nek6 was found to be overexpressed in HCC samples and cell lines by florescent real-time quantitative polymerase chain reaction, immunohistochemistry and western blot analysis. Furthermore, it was evidenced to contribute to oncogenesis and progression. The ectopic overexpression of Nek6 promoted cell proliferation and colony formation, whereas gene silencing of Nek6 inhibited these phenotypes, as documented in Huh7, PLC/PRF/5, Hep3B and HepG2 HCC cell lines. Mechanistic analyses indicated that Nek6 regulates the transcription of cyclin B through cdc2 activation, and promotes the accumulation of G_0_/G_1_-phase cells. In conclusion, the findings of the current study suggested that Nek6 contributes to the oncogenic potential of HCC, and may present as a potential therapeutic target in this disease.

## Introduction

Hepatocellular carcinoma (HCC) is one of the most common types of malignancy globally, with >600,000 mortalities per year, and its incidence continues to increase (1). However, surgery, radiotherapy and chemotherapy have not sufficiently improved the five-year survival rate of patients with this fatal disease in more than three decades. In addition, as HCC is a relatively chemoresistant tumor and highly tolerant to cytotoxic chemotherapy, systemic cytotoxic chemotherapy agents are rarely effective at improving the survival of patients with advanced HCC (2,3). Despite ongoing efforts, no effective biomarkers have yet been identified. Therefore, the development of novel chemotherapeutic agents and more effective therapies for the treatment of HCC are urgently required.

Cell cycle deregulation is one of the hallmarks in human cancer and, thus, identification of physiological targets underlying regulatory mechanisms for cell cycle regulation is critical in the development of novel and effective cancer therapies (4–6). Studies have identified that members of the never in mitosis gene A-related kinase (Nek) family are involved in cell cycle progression (7–9). Neks are essential for mitotic entry, possibly through regulation of the cdc2-cyclin B axis (10). As a member of the Nek family, Nek6 also appears to be involved in cancer. It has been shown that the Nek6 transcript is significantly upregulated in a series of solid tumors, including HCC (11,12). However, the precise molecular mechanism whereby Nek6 contributes to HCC remains unclear.

Despite previous studies which have demonstrated that Nek6 is involved in the oncogenesis of HCC, the potential mechanism remains ambiguous. In the present study, our aim was to investigate the expression of Nek6 in HCC, to explore the role of Nek6 on cell cycle regulation of HCC cells and to trace the internal molecular mechanism.

## Materials and methods

### Cell lines and tissue specimens

The HCC cell lines (huh7, HepG2, Hep3B and PLC/PRF/5) and human normal L02 cells were purchased from Shanghai Cell Bank (Shanghai, China). The cells were incubated in Dulbecco’s Modified Eagle’s Medium supplemented with heat-inactivated 10% fetal bovine serum (Gibco-BRL, Carlsbad, CA, USA) at 37°C in a 5% CO_2_ humidified atmosphere. The 48 pairs of HCC tissues were collected from patients diagnosed with HCC who had undergone liver resection at the First Affiliated Hospital of Nanjing Medical University. The paired normal liver samples were obtained from the same patients, from a region 3 cm away from the edge of the cancer. Patients provided written informed consent and the experiments involving human tissue were proceeded in conformity with the ethical principles of research and approved by the ethics committee of Nanjing Medical University (Nanjing, China).

### Semi-quantitative reverse transcription (RT)-polymerase chain reaction (PCR) and quantitative real-time PCR (qPCR)

Total RNA was extracted using TRIzol reagent (Invitrogen Life Technologies, Carlsbad, CA, USA) according to the manufacturer’s instructions. RT was performed with 2 μg total RNA treated with RNase-free DNase I (Takara Bio, Inc., Shiga, Japan) and the semi-quantitative RT-PCR products were separated on 2% agarose gel containing ethidium bromide. β-actin served as the internal reference. The relative mRNA level was measured by qPCR using SYBR Green I (Takara Bio, Inc.), and the mRNA level of Nek6 in each sample was normalized against β-actin. The primers used were as follows: Forward, 5′-AAGAAGCAGAAGCGGCTCAT-3′ and reverse, 5′-ATGGATCCTCTCCGGTGACA-3′ for Nek6 (249 bp); and forward, 5′-CCTAGAAGCATTTGCGGTGG-3′ and reverse, 5′-GAGCTACGAGCTGCCTGACG-3′ for β-actin (416 bp; loading control).

### Immunohistochemical staining

Nek6 protein expression in the clinical specimens of HCC and non-HCC tissues was determined by immunohistochemistry. The formalin-fixed samples were paraffin-embedded and sectioned (4-μm). The slides were incubated with rabbit anti-human Nek6 polyclonal antibody (1:100 dilution; Santa Cruz Biotechnology, Inc., Santa Cruz, CA, USA) at 37°C for 2 h, where the normal rabbit IgG1 monoclonal antibody (1:100; Santa Cruz Biotechnology, Inc.) was used as a negative control (NC). This was followed by incubation with a horseradish peroxidase-conjugated goat anti-rabbit secondary monoclonal antibody (Dako Japan Co., Ltd., Kyoto, Japan) at 37°C for 1 h. The signals were detected using the diaminobenzidine substrate kit (Vector Laboratories, Burlingame, CA, USA) and counterstaining was performed with hematoxylin.

### Construction of recombinant plasmid

The cDNA for Nek6 was generated by PCR screening of a cDNA library of HCC. The full-length PCR fragment was cloned into the pcDNA3.1-flag expression vector using *Not*I/*Eco*RI. DNA sequencing confirmed the successful construction of the plasmid, and the plasmid for transfection was prepared using a TIANprep mini plasmid kit [Tiangen Biotech (Beijing) Co., Ltd., Beijing, China]. Lipofectamine 2000 transfection reagent (Invitrogen Life Technologies) was used to perform the transfections according to the manufacturer’s instructions.

### Small interfering RNA (siRNA) and short hairpin RNA (shRNA) preparation

For siRNA transfection, oligonucleotides targeting Nek6 were synthesized by Shanghai GenePharma Co., Ltd. (Shanghai, China). The siNek6 sequences used were as follows: 5′-GAUCGAGCAGUGUGACUACdTdT and 5′-GCUCGGUGACCUUGGUCUGdTdT. For shRNA preparation, a synthesized DNA nucleotide fragment encoding shRNA for the knockdown of endogenous Nek6 was inserted into pSUPER (OligoEngine, Seattle, WA, USA). The same vector (pSUPER-shNC) with irrelevant nucleotides not targeting any annotated human genes was used as a NC.

### Cell proliferation and soft agar assay

Cell viability was measured using the Cell Counting Kit-8 (Dojindo Laboratories, Kunamoto, Japan) according to the manufacturer’s instructions. For the soft agar assay, 2,000 cells were cultured in 24-well culture plates containing 1% base agar and 0.5% top agar. The colony morphology was recorded and colony numbers were counted and calculated for each well following the 21-day study period.

### Cell cycle distribution assay

The transfected cells at the logarithmic growth phase were harvested and single-cell suspensions containing 1×10^6^ cells were permeabilized with 70% ethanol. The cells were then labeled with 50 μg/ml of propidium iodide and treated with 250 μg/ml of RNase at 4°C for 30 min. Analysis was performed using FACS Calibur (BD Biosciences, San Jose, CA, USA) and analyzed using Cell Quest software (BD Biosciences). Data are presented as the mean ± standard deviation (SD) from at least three independent experiments.

### Immunoblotting analysis

Western blot analysis was performed according to the manufacturer’s recommended instructions (ImmunoCruz™ IP/WB Optima A System, Santa Cruz Biotechnology, Inc.). Briefly, cell extracts were prepared in lysis buffer [25 mmol/l Tris (pH 6.8), 1% SDS, 5 mmol/l EDTA and protease inhibitor cocktail (1:00); Sigma-Aldrich]. The blot was incubated with blocking solution (5% non-fat milk and 0.1% Tween 20 in phosphate-buffered saline) for 2 h at room temperature. Polyclonal rabbit anti-human Nek6, cdc2 and cyclin B (1:200; Santa Cruz Biotechnology, Inc.) antibodies were used in this study.

### Statistical analysis

Statistical analyses were performed using GraphPad Prism 5 software (GraphPad, La Jolla, CA, USA). Data are presented as the mean ± standard deviation, and were evaluated for statistical significance using unpaired Student’s t-test or one-way analysis of variance, where P<0.05 was considered to indicate a statistically significant difference.

## Results

### Nek6 is frequently upregulated in HCC

**Figure 1:**
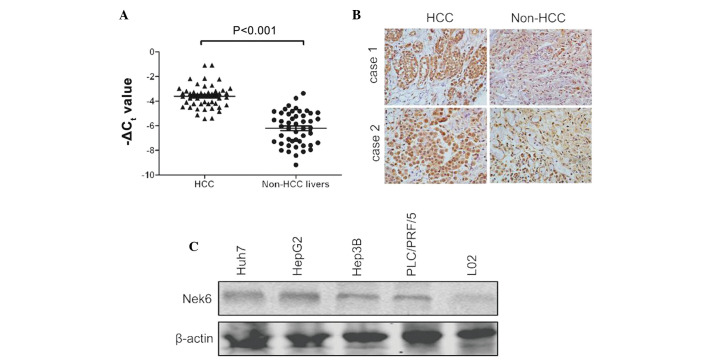
Upregulation of Nek6 in HCC tissues and cells. (A) The expression of Nek6 in 48 paired HCC specimens by real-time polymerase chain reaction. (B) Representative results of upregulated Nek6 in HCC specimens by immunohistochemistry (magnification, ×100; stain, hematoxylin and eosin). (C) Expression pattern of Nek6 in HCC cells and normal L02 cells by western blot analysis. Nek6, never in mitosis gene A-related kinase 6; HCC, hepatocellular carcinoma.

Nek6 was found to be significantly upregulated in 38 (79.1%) of the 48 HCC specimens, whereas the transcript of the gene was rarely detected in adjacent non-cancerous livers, using a semi-quantitative RT-PCR assay (Fig. 1A). To confirm the upregulation of the gene, Nek6 was also evaluated in the 20 pairs of HCC and non-HCC livers through immunohistochemistry. The results showed that the protein levels of Nek6 were evidently increased in HCC when compared with the adjacent non-cancerous livers (Fig. 1B). Nek6 was also markedly expressed in several HCC cell lines, including Huh7, HepG2, Hep3B and PLC/PRF/5 cells (Fig. 1C). Overall, these findings indicated that the upregulation of Nek6 may be a significant event in the oncogenesis of HCC.

### Overexpression of Nek6 promotes cellular proliferation and colony formation

**Figure 2:**
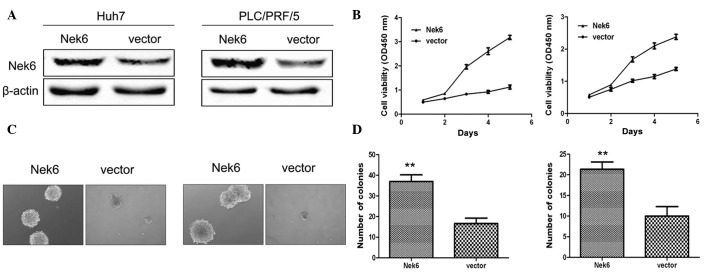
Enforced overexpression of Nek6 promotes the proliferation of HCC cells. (A) The protein level of Nek6 in Huh7 and PLC/PRF/5 cells with pcDNA3.1B-Nek6 transfection. Overexpression of Nek6 (B) enhanced the cell viability and (C) promoted the colony formation of Huh7 and PLC/PRF/5 cells, respectively. (D) Quantification of the colony formation. Data are presented as the mean ± standard deviation of three independent experiments. ^**^P<0.01, vs. the control. Nek6, never in mitosis gene A-related kinase 6; HCC, hepatocellular carcinoma; OD, optical density.

To reveal whether the dysregulated Nek6 may contribute to hepatocarcinogenesis, Huh7 and PLC/PRF/5 cells, which express relatively low levels of Nek6 (Fig. 1C), were transfected with pcDNA encoding Nek6 (Fig. 2A). As compared with the control, exogenous Nek6 promoted the significant cell proliferation of the HCC cells (Fig. 2B). Similarly, ectopic Nek6 exhibited a significantly enhanced effect on the anchorage-independent growth ability of Huh7 and PLC/PRF/5 cells (Fig. 2C and D). These collective results suggested that Nek6 overexpression is significant in promoting the cell growth and colony formation of HCC cells.

### Knockdown of Nek6 inhibits cellular proliferation and colony formation

**Figure 3:**
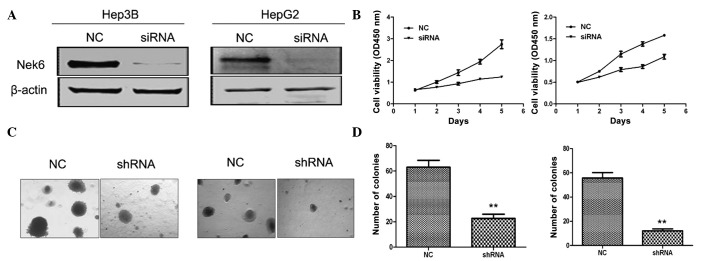
Knockdown of Nek6 inhibits the proliferation of hepatocellular carcinoma cells. (A) The protein level of Nek6 in Hep3B and HepG2 cells with siRNA transfection were evaluated by western blot analysis. The expression of endogenous Nek6 was markedly reduced by Nek6 knockdown. (B) Knockdown of Nek6 inhibited the cell viability of Hep3B and HepG2 cells. (C) Gene silencing of Nek6 weakened the colony formation of Hep3B and HepG2 cells. (D) Quantification of the colony formation. Data are presented as the mean ± standard deviation of three independent experiments. ^**^P<0.01, vs. the control. Nek6, never in mitosis gene A-related kinase 6; NC, negative control; OD, optical density; siRNA, small interfering RNA; shRNA, short hairpin RNA.

To further evaluate the contribution of the upregulation of Nek6 to the oncogenesis of HCC, the siRNA was designed and chemically synthesized for the knockdown of Nek6. To test the efficacy of the siRNA, it was transiently transfected into Hep3B and HepG2 cells, which express relatively high levels of Nek6 (Fig. 1C). The results indicated that the siRNA significantly knocked down the endogenous Nek6, when compared with siRNA-NC (Fig. 3A). Notably, the siRNA evidently inhibited the cell growth of Hep3B and HepG2 cells when compared with the siRNA-NC (Fig. 3B). Furthermore, the shRNA evidently inhibited the colony formation of Hep3B and HepG2 cells in soft agar (Fig. 3C and D). The results suggested that the upregulation of Nek6 may contribute to the hepatocarcinogenesis. Overall, these findings suggested that endogenous Nek6 may be essential for maintaining the cellular proliferation and colony formation of HCC cells.

### Nek6 influences cell cycle progression

**Figure 4:**
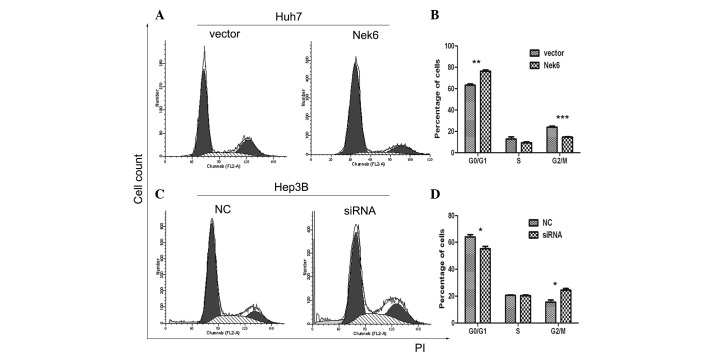
Nek6 influences cell cycle progression. (A) Nek6 overexpression promoted cell cycle progression from G_0_/G_1_ to G_2_/M phase in Huh7 cells. (B) Percentage distribution of cell cycle. Data are presented as the mean ± SD of three independent experiments. ^**^P<0.01 and ^***^P<0.001, vs. the control. (C) Nek6 knockdown induced cell cycle arrest at the G_2_/M phase in Hep3B cells. (D) Percentage distribution of cell cycle. Data are presented as the mean ± SD of three independent experiments. ^*^P<0.05, vs. the control. Nek6, never in mitosis gene A-related kinase 6; NC, negative control; SD, standard deviation; siRNA, small interfering RNA; PI, propidium iodide.

To evaluate the function of Nek6 on cell cycle progression, flow cytometry was performed to detect the cell cycle distribution of Huh7 and Hep3B cells. The enforced Nek6 pushed Huh7 cells to enter into the G_0_/G_1_ phase in advance (Fig. 4C and D); conversely, siRNA targeting endogenous Nek6 led to significant G_2_/M phase arrest, or delayed the entry into the G_0_/G_1_ phase in Hep3B cells (Fig. 4C and D) when compared with the siRNA-NC, which was used as control. These results supported the theory that Nek6 may contribute to the cell proliferation of HCC via promoting the progression of the G_2_/M phase in the cell cycle.

### Nek6 mediates cyclin B transcription through cdc2 modulation

**Figure 5:**
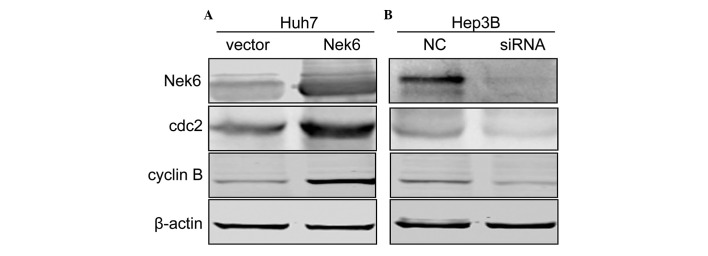
Nek6 mediates cyclin B transcription through cdc2 modulation. (A) Ectopic overexpression of Nek6 in Huh7 cells increased the level of cyclin B through upregulation of the transcription level of cdc2. (B) Knockdown of Nek6 in Hep3B cells reduced the protein level of cyclin B via cdc2 downregulation. Nek6, never in mitosis gene A-related kinase 6; NC, negative control; siRNA, small interfering RNA.

To explore the molecular mechanisms by which Nek6 overexpression contributes to the promotion of the G_2_/M to G_0_/G_1_ transition, specific known important factors were analyzed, including cyclin B, D1 and E, by immunoblotting assay. For Nek6 overexpression, only cyclin B was significantly upregulated in Huh7 cells; by contrast, as Nek6 had been knocked down by siRNA, cyclin B was evidently decreased in Hep3B cells (Fig. 5). This suggested that cyclin B, a key factor responsible for G_2_/M cell cycle phase progression, may be crucial in the promotion of the G_2_/M to G_0_/G_1_ transition that is triggered by Nek6 overexpression. To address the mechanisms responsible for the enhanced cyclin B transcription, the upstream regulatory elements of cyclin B were analyzed. The protein level of cyclin-dependent protein kinase, cdk1/cdc2, was examined using western blot analysis following vector/siRNA transfection. Notably, the protein levels of cdc2 were increased more rapidly in response to Nek6 overexpression (Fig. 5A); however, Nek6 RNAi cells showed clear decreases in cdc2 levels following siRNA transfection (Fig. 5B). These collective results support the hypothesis that Nek6 mediates cyclin B expression in a positive manner through regulation of the transcription of cdc2.

## Discussion

Human Neks have been identified to contribute to cell cycle progression and to be dysregulated in cancer tissues. To date, 11 Neks have been identified in the human genome. Among them, Nek1 (13), 2 (14,15), 10 (16) and 11 (17) are required for G_2_/M arrest. Furthermore, Nek1 is overexpressed in cholangiocarcinoma tumors and MCF7 cells (18,19). Nek8 is upregulated in primary human breast tumors (20), and the ectopic overexpression of Nek10 has been found in breast cancer (21). In HCC, only Nek3 (22) and Nek6 (11,23) have been reported to be upregulated in cancerous tissues.

Nek6 is a serine/threonine kinase belonging to the Nek family, which is significantly involved in mitotic cell cycle progression (9). In a subsequent study, Nek6 was also found to suppress anticancer drug-induced premature senescence (24). Furthermore, it has been shown that Nek6 is able to stimulate tumorigenesis *in vitro* and *in vivo* (25,26). However, the intrinsic functions of Nek6 on tumorigenesis and cell cycle progression in HCC are not well known.

The results of the present study, in conjunction with previous reports (11,23), suggest that the expression of Nek6 is upregulated in HCC tissues compared with the benign normal tissue, which showed low Nek6 expression. The results of the current study also revealed that the enforced Nek6 promoted the proliferation and colony formation of Huh7 and PLC/PRF/5 cells. In addition, Nek6 may be essential for maintaining the hallmark of human HCC cells. Nek6 knockdown also inhibited the cellular proliferation and anchorage-independent growth of Hep3B and HepG2 cells. These results indicated that the upregulation of Nek6 may contribute to oncogenesis and the progression of HCC. To further understand the role of Nek6 in HCC, the cell cycle distribution of HCC cells and the different levels of Nek6 expression were examined. In this study, the ectopic Nek6 promoted the transition from G_2_/M to G_0_/G_1_ phase of the cell cycle, whereas Nek6 knockdown delayed the transition through G_2_/M arrest. Furthermore, Nek6 was found to function as a transactivator of cyclin B via upregulation of the cdc2 level in HCC cells. Cyclin B is a well-documented important regulator that promotes the progression of the G_2_/M phase. This study proposed a possible mechanism in which Nek6 overexpression enhances the upregulation of cdc2, which may in turn activate the cell cycle regulator, cyclin B, in HCC cells in a dominant-positive manner. Subsequently, cyclin B overexpression can promote cell cycle progression and confer specific hallmarks of tumor cells with anchorage-independent growth and tumorigenicity *in vitro*. To the best of our knowledge, this study is the first to uncover the upregulation of Nek6 as an enhancer for cell cycle progression and hepatocarcinogenesis via the promotion of cyclin B expression. However, the complexity of Nek6 contribution to HCC requires further investigation.
